# Positive and Negative Ions Potently Inhibit the Viability of Airborne Gram-Positive and Gram-Negative Bacteria

**DOI:** 10.1128/Spectrum.00651-21

**Published:** 2021-11-10

**Authors:** Sara Comini, Narcisa Mandras, Maria Rita Iannantuoni, Francesca Menotti, Andrea Giuseppe Musumeci, Giorgia Piersigilli, Valeria Allizond, Giuliana Banche, Anna Maria Cuffini

**Affiliations:** a Department of Public Health and Pediatrics, University of Torino, Turin, Italy; University of Mississippi

**Keywords:** air filters, air ionizer, antibacterial activity, *Escherichia coli*, *Staphylococcus aureus*

## Abstract

Positive and negative ions (PAIs and NAIs, respectively) generated by air ionizers curb indoor spread of airborne pathogens through cellular oxidative damage. Thus, here, we asked whether ion exposure of Staphylococcus aureus and Escherichia coli bacteria—either plated on agar or trapped in air filters—would affect their viability and whether this effect would be influenced by variations in bacterial type and load, action area, distance from the ion generator, exposure time, or filter type. We selected these two vegetative bacterium species because, besides being representative of Gram-positive and Gram-negative strains, respectively, they are widely recognized as the two most common airborne pathogens. We observed a robust ion inhibitory effect on the viability of free bacteria regardless of the experimental condition employed. Specifically, 12-h ion exposure of plated S. aureus and E. coli, at either 5 cm or 10 cm from the ion source, reduced bacterial viability by ∼95% and 70%, respectively. Furthermore, 3-h ion exposure was sufficient to reduce the viability of both bacterial species trapped in filters. Our results showing a strong antibacterial activity of PAI and NAI under all experimental conditions tested further support the use of air ionizers for preventing and/or containing airborne infection in domestic and nondomestic settings.

**IMPORTANCE** Indoor air is a well-established vehicle for direct and indirect spread of a wide variety of human pathogens—as bioaerosols are composed of bacteria, viruses, fungi, and other types of organisms—that may trigger some pathologies. Plasmacluster ionizers are known for their ability to generate positively or negatively charged air ions (PAIs and NAIs, respectively) that can kill/inactivate indoor airborne pathogens, through oxidative stress-induced damage, in various environments. Given these premises, the aim of this study was to evaluate the viability of Gram-positive and Gram-negative bacteria exposed to PAI and NAI under different experimental variables such as bacterial type and load, action area, distance from the ion generator, ion exposure time, and filter type. Altogether, our findings, demonstrating a remarkable PAI and NAI antibacterial activity, stress the importance of using air ionizers to prevent indoor airborne infection.

## INTRODUCTION

Ionized air molecules play a major role in keeping the air clean by removing particulates, chemical impurities, and airborne particles of biological origins, known as bioaerosols. Among the most well-established natural sources of ionization are the effects caused by the Earth’s electric field, solar radiations, wind movement, and the splashing of water (1). These energy sources allow the generation of positively or negatively charged air ions (PAIs and NAIs, respectively) whose short life span is influenced by humidity, temperature, and oxygen concentration (2, 3).

Because of our lifestyle habits, we tend to spend a significant amount of time indoors, which renders our bodies highly susceptible to the microbial ecology of indoor environments (4, 5). In this scenario, the risk of indoor transmission of airborne pathogens to our bodies is greatly increased by the tendency of bioaerosols to accumulate in the filters of heating, ventilating, and air-conditioning systems, where these microorganisms can readily multiply under certain environmental conditions. Microbial growth is further enhanced by the availability of organic and inorganic materials deposited on such filters upon air filtration, which favors the release of volatile organic compounds resulting from microbial metabolism. This, in turn, leads to air filter malfunction and deterioration, with possible release of microorganisms into the air (6).

The fact that air ions can inhibit the growth of various airborne microorganisms due to their bactericidal activity (7–9) has brought a renewed interest in the use of air ionizers to control the spread of airborne diseases (e.g., allergies, asthma, and inflammatory lung pathologies) and other infections associated with indoor bioaerosol exposure (7, 10).

The artificial production of small ions by most air ionizers is based on the corona discharge principle, according to which negatively charged ions colliding with suspended particles give the latter a charge so that they can aggregate with each other to form large particles falling out of the air. Thanks to their agglutinating property, these negatively charged ions are even capable of removing bacteria, molds, and viruses from indoor air (9, 11, 12).

In this regard, Plasmacluster ionizers have long been known for their ability to generate PAI and NAI clusters that can kill microorganism through oxidative stress-induced damage (13). Since then, several studies have demonstrated the efficacy of ionizers in disinfecting the air in domestic buildings (6) and car cabins by reducing airborne and surface-adhered microorganisms (5). Ionizers have also been shown to prevent food contamination (14) as well as transmission of hospital-acquired infections (7, 15).

Given the above-described premises, the aim of this study was to evaluate the viability of Gram-positive and Gram-negative bacterial culture exposed to PAI and NAI under different experimental variables such as bacterial type and load, action area, distance from the ion generator, ion exposure time, and filter type.

Our results showing a strong antibacterial activity of PAI and NAI under all experimental conditions tested further support the use of air ionizers for preventing and/or containing airborne infection in domestic and nondomestic settings.

## RESULTS

### Generation of air ions.

The air ion counter revealed that the total ion concentration was 24 and 5 million ions/cm^3^ at 5 and 10 cm, respectively, whereas the average concentration of positive and negative ions emitted by the Plasmacluster ionizer was 12 million ions/cm^3^ at a 5-cm distance and 2.5 million ions/cm^3^ at a 10-cm distance (Table 1). Neither the room temperature (RT) (25 ± 2°C) nor the relative humidity (RH) (50% ± 10%) interfered with ion production.

**TABLE 1:** Emitted concentrations of NAIs and PAIs

	Plasmacluster ionizer	
	Distance from petri dishes (cm)	
Ion count (million ions/cm^3^)	5	10
Total positive ions	12.3	2.9
Total negative ions	11.7	2.1
Avg total concn of positive and negative ions	12	2.5

### Direct effects of air ion exposure on bacteria under different experimental conditions.

**FIG 1:**
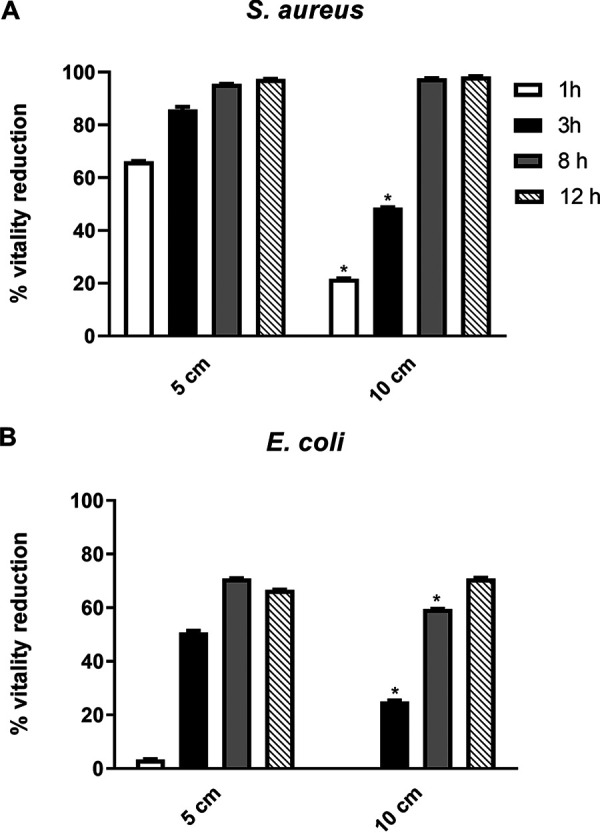
(A and B) Direct ion effect on the viability of S. aureus (A) and E. coli (B) plated at 10^4^ CFU/ml on 150-mm petri dishes, with the ionizer being placed at a distance of 5 or 10 cm. *n* ≥ 3 replicate experiments. *, *P* < 0.05, Student’s *t* tests.

**10^4^ CFU/ml plated bacteria.** In this series of experiments, 10^4^ CFU/ml of Staphylococcus aureus and Escherichia coli, both plated on 150-mm petri dishes, were exposed for the indicated times to the ions emitted by a Plasmacluster ionizer, as described in detail in Materials and Methods. After 3 h of ion exposure at 5 or 10 cm from the ion source, the viability values of S. aureus were reduced by approximately 86% and 49%, respectively (*P < *0.05) (Fig. 1A). Likewise, albeit to a lower extent, ion exposure of E. coli, under the same experimental conditions, resulted in a decreased viability of about 51% and 25%, respectively (*P < *0.05) (Fig. 1B). After 8 to 12 h of ion exposure, regardless of the distance from the ion source, a further reduction in bacterial viability resulted, reaching values of >95% for S. aureus (Fig. 1A) and 60 to 70% for E. coli (Fig. 1B). Intriguingly, the difference in reduction of bacterial viability between the two distances (5 versus 10 cm) was only significant (*P < *0.05) at the 3-h time point, while it gradually decreased at 8 h and was virtually undetectable at 12 h, indicating that long-distance ion exposure can still exert a bactericidal activity, provided that the exposure time is adequate (Fig. 1). Similar results were obtained when the same bacteria were spread on 90 mm petri dishes (data not shown).

**10^7^ CFU/ml plated bacteria.** When we plated bacteria at a concentration of 10^7^ CFU/ml, due to the patina growth of the control plate, which did not allow colony counting, we were unable to obtain an exact viability reduction percentage. Nevertheless, visual examination of the petri dishes at all time points seemed to indicate an inhibitory effect of ion exposure at either distance (5 and 10 cm) on the growth of S. aureus plated on 150- and 90-mm petri dishes (see Fig. S1A in the supplemental material and data not shown, respectively). In contrast, under the same experimental conditions, we only detected a slight ion effect on E. coli growth (Fig. S1B), mainly after 8 and 12 h of ion exposure. Of note, the majority of the surviving bacteria were located on the outer edges of the petri dishes, where the ion concentration is supposed to be lower than in the central area, which is directly beneath the point electrodes (Fig. S1A and B).

### Ion effects on bacteria soaked in air filters under different experimental conditions.

**FIG 2:**
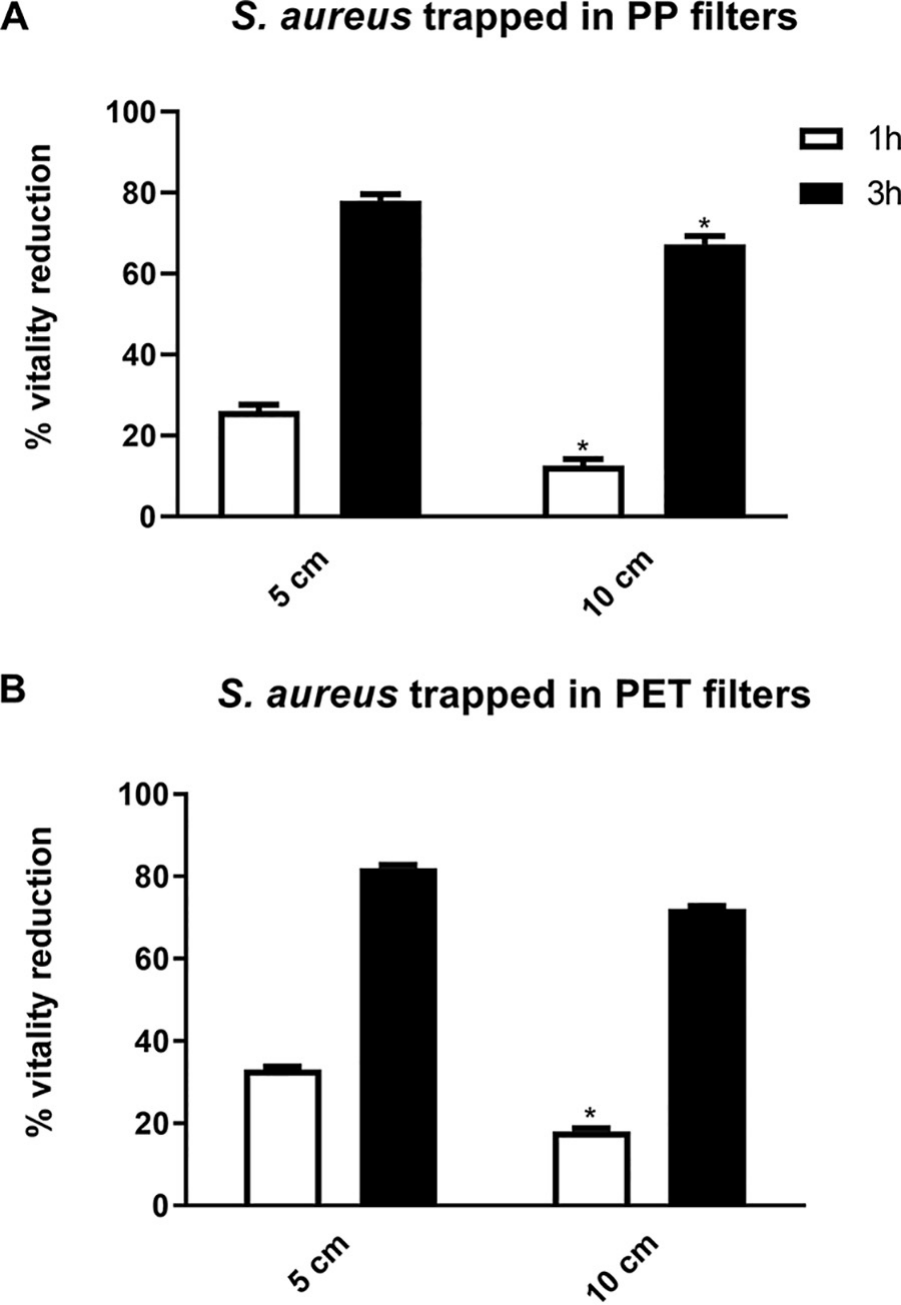
(A and B) Ion effect on the viability of S. aureus trapped in PP (A) or PET (B) filters at 10^4^ CFU/ml, with the ionizer being placed at a distance of 5 or 10 cm. *n* ≥ 3 replicate experiments. *, *P* < 0.05, Student’s *t* tests.

**FIG 3:**
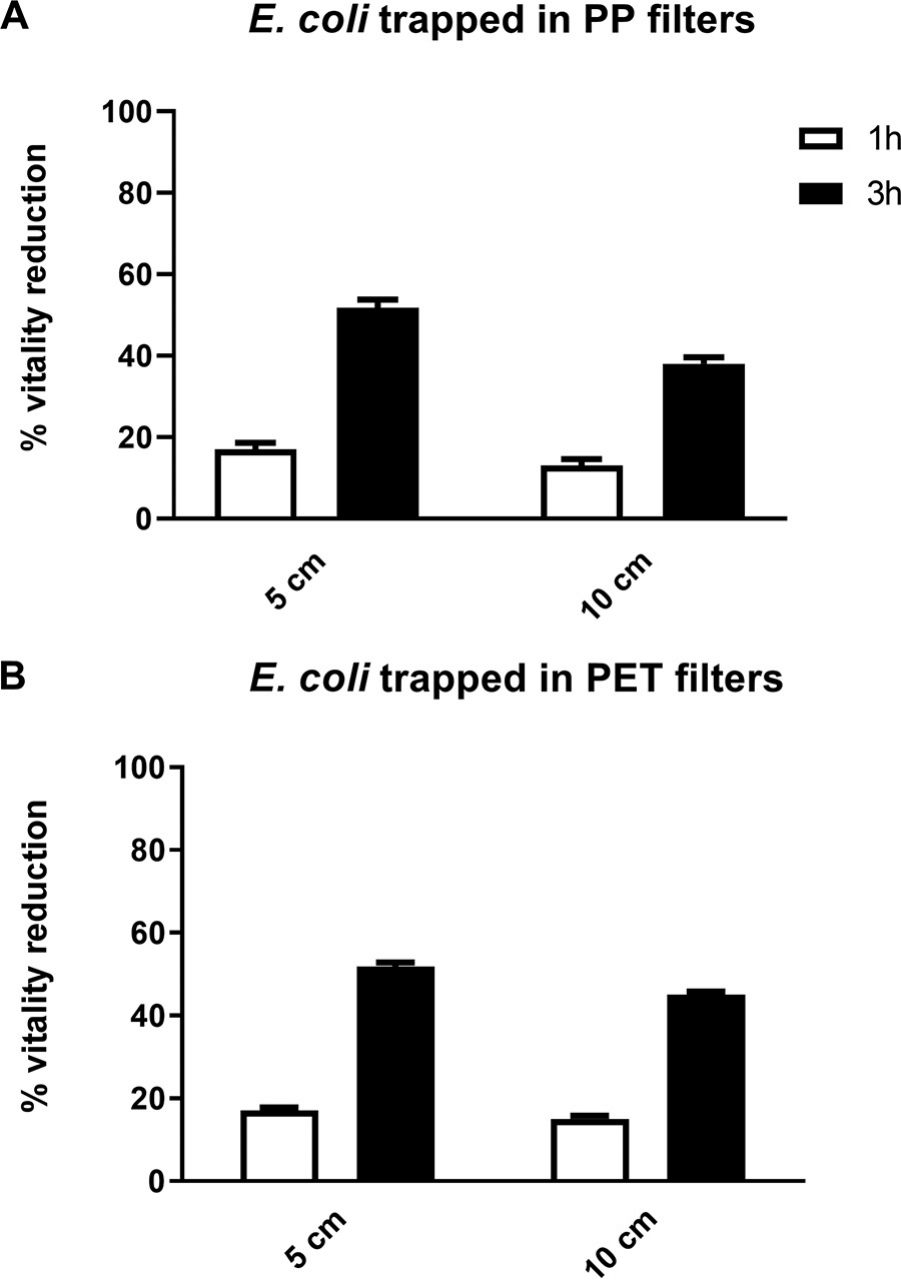
(A and B) Ion effect on the viability of E. coli trapped in PP (A) or PET (B) filters, at 10^4^ CFU/ml, with the ionizer being placed at a distance of 5 or 10 cm. *n* ≥ 3 replicate experiments.

In these experiments, two types of filters were used, polypropylene (PP) filters or Combi filters in PP and polyethylene terephthalate (PET), the latter containing activated carbon particles. PP or PET filters soaked with S. aureus and E. coli at 10^4^ CFU/ml were exposed to ions for the indicated times. After 1 h of ion exposure, we observed a slight inhibitory effect on both strains under all experimental conditions, although the differences in viability reduction were not statistically significant (Fig. 2 and 3). After 3 h of ion exposure at 5 cm and 10 cm from the ion source, we recorded a substantial reduction in S. aureus viability, with values ranging from 78 to 67% on PP filters (*P < *0.05, Fig. 2A) and from 72 to 82% on PET filters (Fig. 2B), respectively. Similarly, 3-h ion exposure at a distance of 5 and 10 cm reduced the viability of E. coli by, respectively, 52% and 37% on PP filters (Fig. 3A) and 52% and 45% on PET filters (Fig. 3B). A similar reduction in bacterial viability was observed when we tested S. aureus and E. coli at a concentration of 10^7^ CFU/ml (data not shown).

## DISCUSSION

Indoor air is a well-established vehicle for direct and indirect spread of a wide variety of human pathogens (16–18), as bioaerosols are composed of bacteria, viruses, fungi, and other types of organisms (19). Generally, most airborne bacteria and fungi do not affect healthy humans, but they may trigger asthma, allergies, or infections in susceptible individuals, such as young children, the elderly, and immunocompromised individuals (19–22).

Over the years, multiple engineering solutions have been developed to remove and/or inactivate indoor airborne pathogens in domestic and nondomestic environments. These include air ionization, air filtration, UV germicidal irradiation, and dielectric barrier discharge (18, 19). The control and containment of indoor airborne pathogens is particularly important in health care settings, where for instance, poor operating room conditions may influence the outcome of a surgical intervention, affect patient safety, influence operating team comfort, and produce suboptimal clinical conditions. It is generally accepted that high levels of airborne microbial contamination are responsible for increased infection rates in surgical sites, although no direct association between airborne microbial contamination and surgical wound infection has been formally demonstrated (23). Furthermore, in all health care facilities, including ambulances, there exist several sources of infectious agents, such as patients, staff, and the environment itself once it becomes contaminated (20).

With regard to other types of closed environments, Luksamijarulkul et al. have shown that drivers of air-conditioned buses are especially at risk of airborne and droplet infections due to the unhygienic conditions of the air and poor ventilation inside the bus cabin (24, 25). Furthermore, several studies have documented airborne microbial transfer in food production environments. Indeed, the microbiota present in the air has the potential to cause foodborne disease and reduce the shelf life of food products, leading to potential food safety issues and economic losses for the food industry. In food facilities, good levels of hygiene are generally reached by implementing a sanitation process capable of eliminating most pathogenic and spoilage microorganisms, since a completely sterile environment is unrealistic and unnecessary (26).

In light of the above-described issues, the purpose of the present study was to assess the inhibitory effect of PAIs and NAIs on the viability of S. aureus and E. coli, plated on agar or soaked in air filters, under different experimental conditions—i.e., bacterial load, action area, distance from the ion generator, exposure time, and filter types. We selected these two vegetative bacterium species because, besides being representative of Gram-positive and Gram-negative strains, they are widely recognized as the two most common airborne pathogens. To achieve a more precise direct count of only surviving bacteria, we decided to use the same methodology described in the literature (8, 11, 27, 28), which consisted in the spread of an exact bacterial inoculum on agar subjected to the ion treatment. Finally, to better compare the results obtained here with those of other authors in the field, we expressed the results as percentage values (8, 27).

Our results provide clear evidence of an inhibitory activity of ions on the growth of free bacteria under all the experimental conditions tested, and it should be underscored that the emitted ion concentration does not negatively affect human health (29); rather, in a recent study by Jiang et al. (9) a beneficial action of NAI has been revealed, mainly in relieving respiratory symptoms. In particular, when both species were plated at a density of 10^4^ CFU/ml, we recorded similar percentages of viability reduction at all time points of ion exposure. Remarkably, at the 12-h time point, the percentage of reduction in bacterial viability was greater than 95% for S. aureus and about 70% for E. coli, regardless of the distance of the petri dishes from the ion source (5 versus 10 cm). These findings are in good agreement with a study by Park et al. (8) showing that PAI- and NAI-induced oxidative stress causes extensive death of S. aureus, Enterococcus faecalis, and Bacillus subtilis species. Congruently, Tyagi et al. (27) showed a temporal relationship between NAI exposure time and reduction in E. coli viability. Other studies documented the bactericidal effects on E. coli of both negative- and positive-polarity electrical discharges in nitrogen (8). Finally, Noyce and Hughes (28) were the first to characterize the ionic component of disinfection by electrical discharges.

Another important finding of our study is that ion exposure promoted a robust growth-inhibitory effect on both bacterial strains plated or soaked at two concentrations (i.e., 10^4^ or 10^7^ CFU/ml), though, as expected, it was slightly less marked when bacteria were loaded in a larger quantity. The high inoculum testing (10^7^ CFU/ml) was performed to assess the ion effect on a soiled condition. In addition, the observation that the reduction of bacterial viability was also achieved in 150-mm petri dishes suggests that ions have a quite wide action range, not solely limited to the area covered by the air ionizer.

Considering the exposure time, we observed an antimicrobial effect as early as after 1 h of ion exposure that became increasingly significant over time (3 and 12 h), suggesting that prolonged use of ionizers may lead to better air purification.

Finally, considering the distance from the ion source, we found that the antimicrobial activity partly depends on the distance between the electrodes of the ionizers and the plated bacteria. A significant growth inhibition was, in fact, recorded at both distances (5 cm and 10 cm), although, as expected, at 10 cm the viability reduction percentages were lower.

Even though a growing number of studies have focused on the role of PAIs or NAIs in promoting inactivation or growth inhibition of various bacterium strains, to date, the physical and biological mechanisms underlying this effect remain unclear (7, 8). Several hypotheses have been formulated to explain the bactericidal action of ionizers, including electrical phenomena due to ozone production (30), electrodynamics effects of negative and positive ions (8), electrostatic repulsion (31), and electroporation mechanism (7).

The biocidal activity of ions, shown here, is in keeping with previously published data (7, 27, 28, 32). Our findings, in accordance with the available literature, speculate that the bactericidal effect may be due to oxidative damage driven by the generation of reactive oxygen species (ROS) in response to corona discharges, which ultimately may affect bacterial biomolecules, such as lipids, proteins, and DNA (33–36). These reactions may also alter some intrinsic bacterial membrane properties such as fluidity, ion transport, protein cross-linking, and site-specific amino acid modifications. In addition, they may inactivate certain enzyme activities, inhibit protein synthesis, oxidize DNA, cause double-strand breaks, and remove nucleotides, thereby leading to cell death (37). In the present work we did not did attempt to eliminate the effects of ozone; thus, as reported by Fletcher et al., the possible explanation of the bactericidal effect of the generated ions appears to be oxidation damage arising from exposure to ozone produced by the electric discharge (7). Thus, we can hypothesize that the differences in viability decrease observed here between S. aureus and E. coli might be due to the different compositions of the external layers of the bacteria, rendering a Gram-negative bacterium less susceptible to the ion effect than a Gram-positive one.

Relevant to this study is the tendency of bioaerosols to accumulate in large quantities also on the filters of heating, ventilating, and air-conditioning systems, where they can multiply under certain conditions, especially when lots of moisture is present on the filters. Moreover, the organic or inorganic material deposited on the filter medium upon air filtration favors microbial growth. This inevitably hampers filter efficacy, possibly leading to deterioration followed by the release of more microorganisms into the air (19). This has led us to determine whether ion exposure would inhibit the viability of bacteria trapped in air filters and whether such an effect would be influenced by variations in experimental conditions such as bacterial type and load, distance from the ion generator, exposure time, and filter type. We show a robust antibacterial activity of ions against both bacterial species trapped in the filters as early as after 3 h of ion exposure, independent of concentration and distance from the ionizing source. Ion exposure times of 8 h and 12 h were not evaluated on filter-trapped bacteria since the use of the ionizer was hypothesized to improve the microbiological quality of air in closed rooms for short time. Furthermore, the presence of activated carbon in the Combi filters did not appear to affect the antibacterial activity of the ions. Although data in the literature have shown ions to be less effective against higher bacterial loads, here, we recorded a very good antimicrobial effect even on bacteria at a concentration of 10^7^ CFU/ml, a condition difficult to find in indoor systems, where filters tend to be washed or replaced. These results are in good agreement with those of Kim et al. (6), showing an antibacterial activity of PAIs against aerosolized E. coli and S. epidermidis collected on membrane filters.

Despite the limitation of the present study, mainly ascribable to the use of only two strains representative of Gram-positive and Gram-negative bacteria, to the best of our knowledge, this is the first study that characterizes the ion effectiveness in reducing the viability of bacteria according to concentration, length of exposure, and distance from the ion source.

Overall, our findings can provide the rationale for the use of ion air purifiers to prevent and/or contain infection in health care and other settings. Experiments are under way to test whether this air sanitation approach is suitable for other airborne infectious agents, such as fungi, mycobacteria, and viruses.

## MATERIALS AND METHODS

### Air ion generation.

Air ions were generated by means of a Plasmacluster ionizer (DENSO Thermal Systems SpA, Poirino, Turin, Italy). This device produces a balanced cluster of positive hydrogen ions (H^+^) and negative oxygen ions (O_2_^−^) from the water and oxygen in the air through plasma discharge, a process during which voltage is applied to a discharge electrode. The ionizer was placed on a support to reach a distance of 5 cm and 10 cm from the petri dishes or the air filters.

### Ion capture.

PAI and NAI emitted concentrations were measured with an air ion counter (AlphaLab, Inc., Salt Lake City, UT, USA). This device is a handheld meter designed to measure separately the densities of PAIs and NAIs, expressed as number of ions per cubic centimeter (ions/cm^3^). It contains a fan that pulls air through the meter at a calibrated rate. Air is sucked in at the top of this instrument, measured, and ejected at the bottom. The display shows the ion count, and it continues to display the ion density in the air, showing any changes.

### Bacteria.

S. aureus ATCC 29213, as a representative Gram-positive bacterium, and E. coli ATCC 25922, as a representative Gram-negative bacterium, were cultured at 37°C on Mannitol salt agar (MSA; Merck, Darmstadt, Germany) and MacConkey agar (MAC; Sigma-Aldrich, Milan, Italy), respectively. After 18 to 24 h of incubation, colonies were inoculated into cryovials—containing porous beads and a cryopreservative fluid—and maintained at −80°C for extended storage (38–43). Bacterial cultures were serially diluted in 0.9% NaCl saline solution (Baxter S.p.A., Rome, Italy) to obtain two inoculum concentrations, 10^4^ and 10^7^ CFU/ml, confirmed by plating them on nutrient agar medium (NA; Sigma-Aldrich).

### Filters.

Two filter types were used, particle filters in polypropylene (PP) and Combi filters in PP and polyethylene terephthalate (PET); only the latter type was filled with activated carbon particles. The materials were cut to fit a 5-cm by 2.5-cm face filter, packaged in waxed paper, and sterilized in an autoclave at 121°C for 15 min.

### Microbiological assay set up.

**FIG 4:**
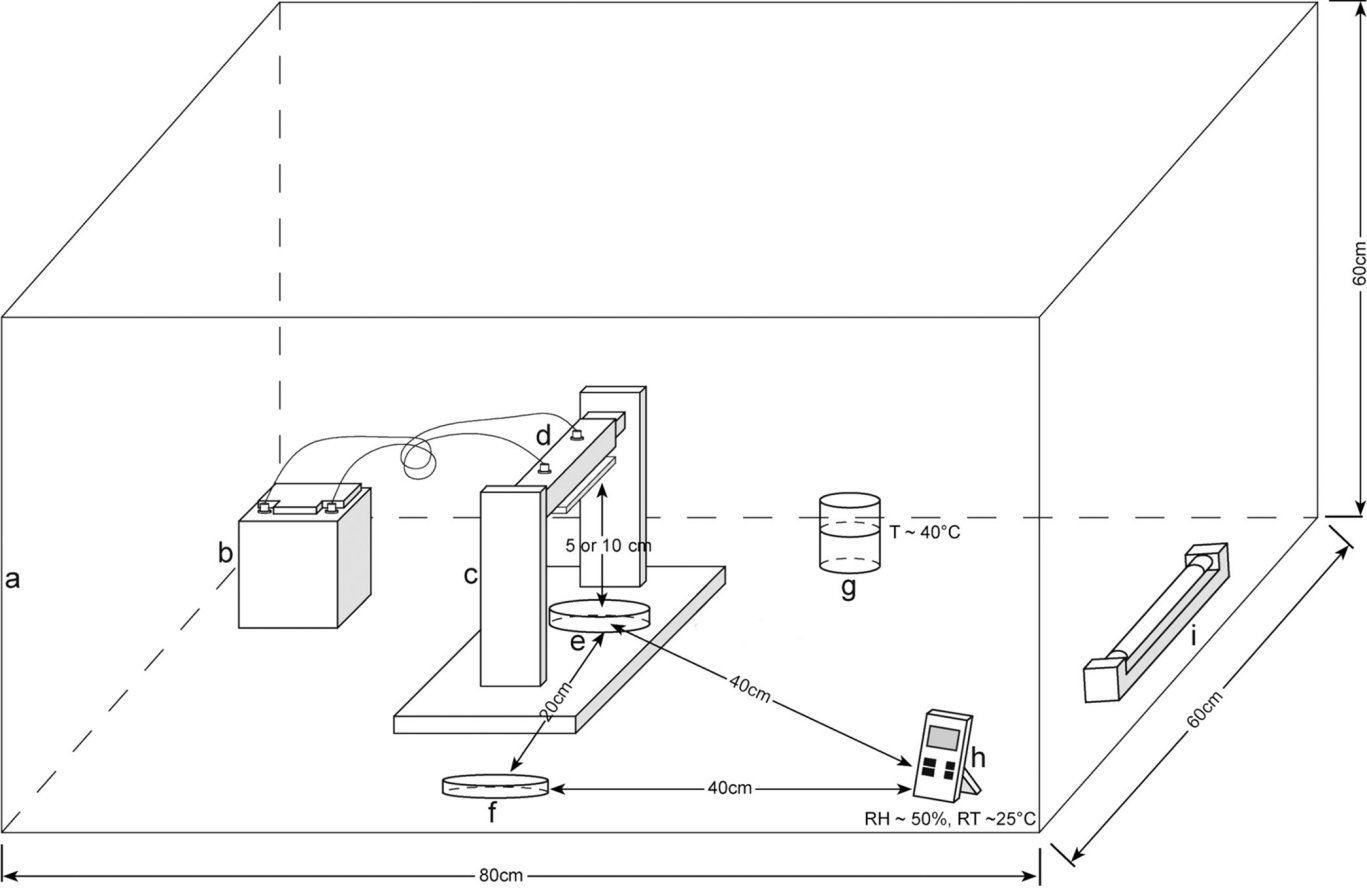
Schematic representation of the ionization setup. (a) vertical flow cabinet; (b) battery; (c) ionizer support; (d) ionizer; (e) NA petri dish with seeded bacteria or empty petri dish with bacteria trapped into filters exposed to ions; (f) NA petri dish with seeded bacteria or empty petri dish with bacteria trapped into filters not exposed to ions (controls); (g) container with hot water to keep a constant humidity; (h) humidity/temperature data recorder; (i) UV lamp.

All ion exposure experiments were performed under aerobic sterile conditions at room temperature (RT, ∼25°C) and about 50% relative humidity (RH) in a closed and turned off laminar flow cabinet. Temperature and RH were monitored with a humidity/temp data recorder (PCE Instruments, Alicante, Spain) (Fig. 4).

**Direct exposure of bacteria to PAIs and NAIs.** The direct ion effects on bacteria plated on agar were evaluated under the following experimental conditions: (i) using Gram-positive (S. aureus) and Gram-negative (E. coli) bacterial culture, (ii) assaying two bacterial concentrations (10^4^ and 10^7^ CFU/ml), (iii) plating bacteria on two diameters of NA petri dishes (90 and 150 mm), (iv) placing the bacterial NA petri dishes at two distances from the ion source (5 and 10 cm), and (v) exposing bacteria to ions for different time periods: 1, 3, 8, and 12 h.

Each bacterial dilution was spread on NA petri dishes, placed without a lid under the ionizer, and exposed to ions as described above. As a negative control, similarly plated bacteria were grown in parallel without being exposed to ions. After being exposed to ions for the indicated times, all petri dishes were incubated at 37°C for an additional 18 to 24 h to allow the CFU count. All experiments were repeated at least three times.

**Exposure of filter-trapped bacteria to PAIs and NAIs.** The effects of ion exposure on filter-trapped bacteria were evaluated under the following experimental conditions: (i) using Gram-positive (S. aureus) and Gram-negative (E. coli) bacterial culture, (ii) assaying two bacterial concentrations (10^4^ and 10^7^ CFU/ml), (iii) using two air filter types (PP and PET), (iv) placing the filters at two distances from the ion source (5 and 10 cm), and (v) exposing bacterium-containing filters to ions for different time periods (1 and 3 h).

Both air filter types were separately immersed in sterile tubes containing S. aureus and E. coli at two inoculum concentrations and incubated for 3 h at 37°C to allow the bacterial culture to deposit on the filters. The filters were then removed from the tubes, positioned on empty sterile petri dishes without lids, and exposed or not (negative control) to ion treatment under aerobic sterile conditions at RT and constant RH, as described above. After being exposed to ions for the indicated time periods, the filters were placed in tubes containing 0.9% NaCl saline solution and sonicated (35 kHz; Bandelin Sonorex Digitec; Bandelin Electronic GmbH & Co. KG, Berlin, Germany) for 10 min to detach bacteria from the filters without affecting their viability (39, 40, 43). After sonication, the filters were removed, and the tubes were centrifuged at 4,000 rpm for 10 min. The ensuing pellets were resuspended and serially diluted in saline solution. Each dilution was spread on NA medium and incubated at 37°C for 18 to 24 h to allow the CFU count. All experiments were repeated at least three times.

### Statistical analysis.

Data were expressed as a percentage variation with respect to the control according to the following formula:

$$\text{\% Viability Reduction}=\left(\frac{X_{f}-X_{i}}{X_{i}}\cdot 100\right)$$

Where *X_f_* indicates the number of colonies after ion exposure, and *X_i_* indicates the number of colonies of the respective growth control.

Statistical analysis was carried out using the Prism version 9 for Windows (GraphPad Software, San Diego, CA, USA). For comparisons consisting of two groups, means were compared using two-tailed Student’s *t* tests. Differences were considered statistically significant at a *P* value of <0.05.
