# Supplementary material for: Positive and Negative Ions Potently Inhibit the Viability of Airborne Gram-Positive and Gram-Negative Bacteria
Source: Microbiol Spectr. 2021 Nov 10;9(3):e00651-21. doi: 10.1128/Spectrum.00651-21 (PMC8579920; doi:10.1128/Spectrum.00651-21)

Figure S1. *Direct ion effect on the viability of S. aureus (**A**) and E. coli (**B**) plated at 10^4 CFU/ml on 150 mm Petri dishes, with the ionizer being placed at a distance of 5 or 10 cm. * $P < 0.05$.*

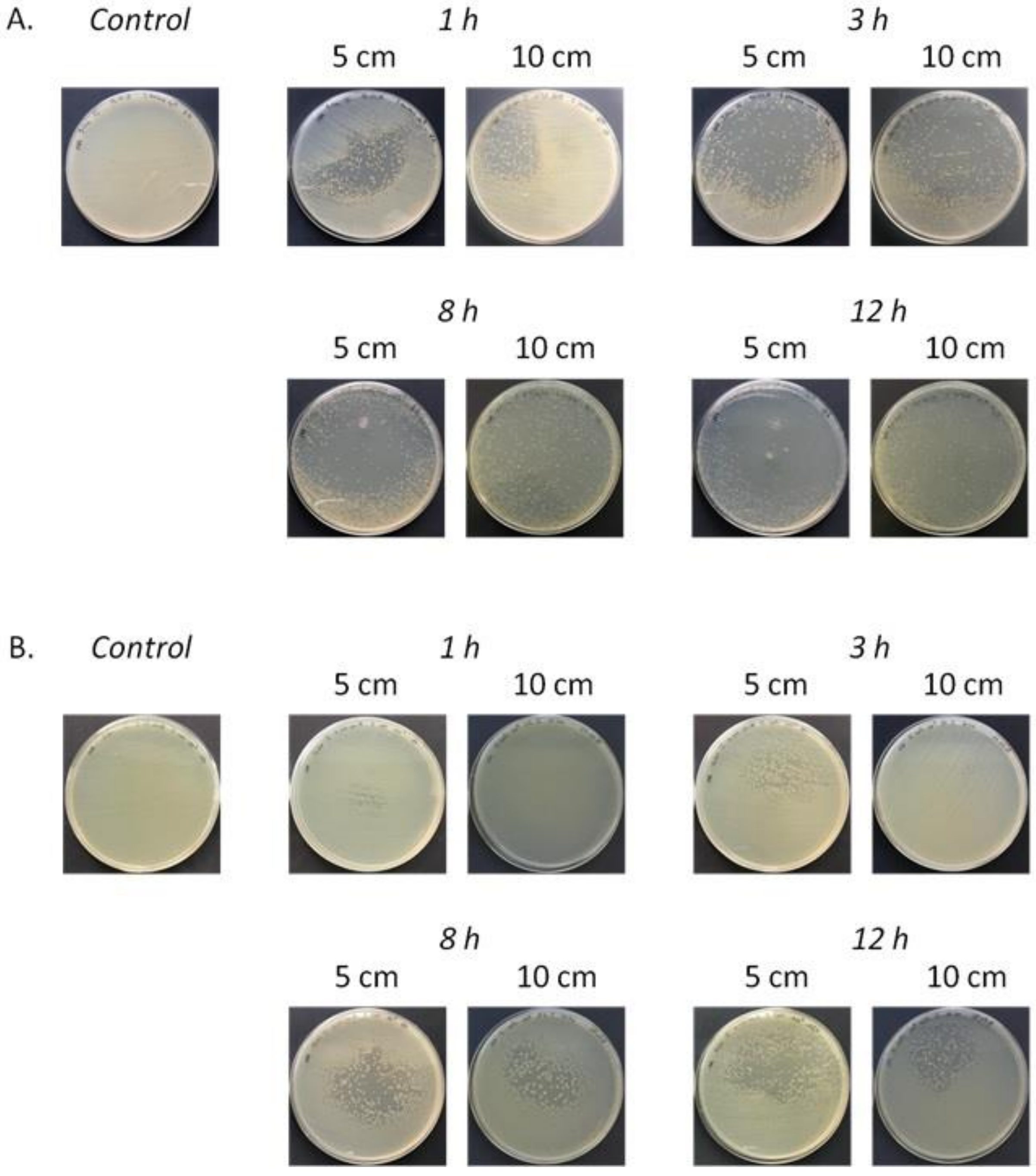

Supplement: SUPPLEMENTAL FILE 1 — Supplemental material. Download SPECTRUM00651-21_Supp_1_seq2.pdf, PDF file, 0.1 MB [file spectrum00651-21_supp_1_seq2.pdf]
